# Association between *FADS* Gene Expression and Polyunsaturated Fatty Acids in Breast Milk

**DOI:** 10.3390/nu14030457

**Published:** 2022-01-20

**Authors:** Huimin Tian, Haitao Yu, Yiqi Lin, Yueting Li, Wenhui Xu, Yiru Chen, Guoliang Liu, Lin Xie

**Affiliations:** 1 Department of Pediatric Nursing, School of Nursing, Jilin University, Changchun 130021, China; tianhm@jlu.edu.cn; 2 Department of Nutrition and Food Hygiene, School of Public Health, Jilin University, Changchun 130021, China; htyu19@mails.jlu.edu.cn (H.Y.); ytli20@mails.jlu.edu.cn (Y.L.); xuwh20@mails.jlu.edu.cn (W.X.); yrchen20@mails.jlu.edu.cn (Y.C.); glliu@jlu.edu.cn (G.L.); 3 Jilin Women and Children Health Hospital, Changchun 130061, China; linyiqi791222@126.com

**Keywords:** breast milk, polyunsaturated fatty acid, *FADS* gene, gene expression

## Abstract

Polyunsaturated fatty acid (PUFA) in breast milk provides physiological benefits for offspring and is closely related to endogenous biosynthesis in lactating women. Few studies have addressed the association between fatty acid desaturase (*FADS*) gene expression patterns and fatty acids in breast milk. This research aimed to explore the differences in PUFA levels among breast milk groups with different levels of *FADS* gene expression and provide a scientific basis for precision nutrition strategies. A total of 50 healthy women 42–45 days postpartum were included in this study. A basic information questionnaire and breast milk samples were collected. Eight types of PUFA were detected, and RNA was extracted from breast milk. The transcription level of the *FADS* gene was detected using real-time quantitative PCR. Significant differences in the content of gamma-linolenic acid and eicosatrienoic acid (C20:3n6) were found in breast milk among *FADS1* gene transcription groups (*p* = 0.009, *p* = 0.042, respectively). No significant differences in PUFA were found among the *FADS2* and *FADS3* gene expression groups. The results demonstrated that n-6 PUFA was associated with the mRNA expression levels of the *FADS1* gene. They are of great significance in developing new methods and diets to optimize infant feeding using breast milk.

## 1. Introduction

Breast milk, known as “white blood”, provides critical nutrients such as beneficial microbes, and it is an ideal food source for breastfeeding babies [1]. The macronutrients in human breast milk such as carbohydrates, proteins, and fats, are a source of energy, and approximately 50% of this energy is derived from fatty acids (FAs) [2]. Studies have shown that the macronutrients present in breast milk are relatively consistent among lactating women [3], while FA content varies greatly [2,4,5].

FAs in breast milk are classified into saturated FA (SFA), monounsaturated FA (MUFA), and polyunsaturated FA (PUFA) according to the number of unsaturated double bonds in their chemical structure. Furthermore, n-3-series and n-6-series FAs exist in PUFA according to the position of their unsaturated double bonds [6]. These three types of FAs play different roles in the body. Dietary SFA is a critical factor that seems to trigger alterations associated with the function of insulin resistance [7]. A diet rich in MUFA could reduce the number of established disease biomarkers in patients, as well as the incidence of coronary heart disease and type 2 diabetes [8]. In contrast, PUFA, in particular eicosapentaenoic acid (EPA) and docosahexaenoic acid (DHA), plays a beneficial physiological role in offspring receiving this FA during critical periods of development [9]. It is well known that DHA plays an important role in the growth and development of the brain and retina in infants [10,11,12]. EPA and DHA in breast milk also have beneficial effects on anti-inflammation [13], infant psychomotor development [14,15], and reduce the risk of childhood allergic disease [16].

Research shows that the postnatal development of an infant is strongly associated with the FA content of their mother’s milk during lactation, and this FA profile may influence the individual risk of developing metabolic diseases throughout their life [9,17]. The World Health Organization pointed out that infants should be exclusively breastfed for more than 6 months from birth, and after 6 months, supplementary food may be added while maintaining breastfeeding until the age of 2 years old or more [18]. In addition, the first 2 years following birth represent a critical period for the development of the brain and retina in infants and young children [19]. PUFA in breast milk is the only dietary source for exclusively breastfeeding infants. Therefore, ensuring sufficient levels of PUFA in breast milk is essential for the long-term growth and physical health of babies.

A systematic review and meta-analysis indicated significant variation in the FA profiles in breast milk among different populations [20]. The intake of deep-sea fish and shrimp among inland lactating women was lower than that in mothers living in other areas. Therefore, their level of PUFA intake, as well as the content of DHA in their bodies, was lower [21]. Furthermore, in a population from the same area, the composition of PUFA in the breast milk of lactating women was closely related to endogenous biosynthesis in addition to dietary intake [22]. Thus, it is more critical to study the synthesis of PUFA in the bodies of lactating mothers living in inland areas where the dietary intake of fish is lower.

Two key enzymes are present in the bioconversion of the essential FAs linoleic acid (LA) and α-linolenic acid (ALA) into longer-chain PUFA: Δ-5 desaturase and Δ-6 desaturase. Δ-5 desaturase is encoded by the *FADS1* gene, while the *FADS2* gene is responsible for Δ-6 desaturase. However, another *FADS3* gene is located in the *FADS* gene cluster, and its function is uncertain [23]. Research on the association between PUFA and *FADS* gene expression has mainly focused on animals. In animal experiments, it was found that the mRNA expression levels of *FADS1* and *FADS2* genes in rats fed with lower PUFA levels were significantly increased [24]. Furthermore, most studies have been performed on human tissue derived from the blood and liver, while few studies have evaluated the association of breast milk composition in the context of the *FADS* gene [25,26]. *FADS* gene expression has tissue-specific effects [25], and it is necessary to study the relationship between PUFA and *FADS* gene expression in breast milk to understand PUFA regulation.

The cellular components of human milk offer the possibility to study the association between the expression of *FADS* gene clusters and PUFA constituents in breast milk [27]. In this study, fresh breast milk was used as the sample, and molecular biology technology was applied to detect the mRNA expression of the *FADS* gene cluster. This study focused on the association between FADS gene expression and PUFA profile in the milk of lactating mothers living in inland areas. We hypothesized that the higher the mRNA level of the *FADS* gene expressed among lactating women, the higher the proportion of PUFA contained in their breast milk. The purpose of this research was to explore the differences in PUFA levels in relation to different *FADS* gene expression groups and provide a scientific basis for optimizing the management of infant feeding.

## 2. Materials and Methods

### 2.1. Participants

This study was conducted from January 2017 to August 2017 and was a cross-sectional survey. Participants in this study were from a women and children health care hospital in Changchun City, Jilin Province, China, including a total of 50 Chinese Han lactating mothers 42–45 days postpartum. The eligibility criteria were: a healthy, breastfeeding woman without any diseases during pregnancy and lactation; not taking PUFA supplements after delivery; and a healthy, singleton infant with a gestational age of ≥37 weeks and a weight ≥2500 g. The study procedure was approved by the Ethics Committee of the School of Public Health, Jilin University, China (No. 2016-08-01). All mothers provided informed consent at the beginning of this research.

### 2.2. Breast Milk Collection and Questionnaires

We collected breast milk samples from each volunteer by manual expression, and a total of 20 mL of breast milk was placed into two clean tubes between 9:00 and 11:00 a.m. One tube of breast milk was used to detect PUFA, while the other tube was used to extract RNA. All breast milk samples included mature milk and were quickly positioned in a 4 °C icebox. Samples were stored in a −80 °C refrigerator, and detection took place within 2 weeks. A questionnaire was used to investigate each participant’s basic information, and it was implemented through face-to-face interviews by trained investigators preceding milk collection.

### 2.3. Fatty Acids Analysis

PUFA in breast milk was detected using capillary gas chromatography, and the protocol of the experiment was described in detail in previous studies [28,29]. A total of eight types of PUFA in breast milk were detected, including four n-6 PUFA and four n-3 PUFA. The level of PUFA in breast milk was presented using the weight percentage of each FA in total FA.

### 2.4. RNA Extraction

The TRIzol method was used to extract total RNA from breast milk. The tubes containing 10 mL of breast milk were removed from the −80 °C freezer and thawed at room temperature. Liquid milk was immediately centrifuged at 4 °C, and three liquid levels were clearly detected: the upper semifluid was the fat in breast milk, the intermediate liquid was skimmed breast milk, and the cells were at the bottom of the tube [30]. The precipitated cells were kept at the bottom of the tube, and 1 mL of phosphate-buffered saline (PBS) was added to the tube to wash them. Then, the PBS containing precipitated cells was transferred into a new 1.5 mL tube and centrifuged for 15 min. The supernatant was discarded, and 1 mL of TRIzol reagent (Takara, Japan) was added to the tube containing the precipitate. Afterward, the tube was left at room temperature for 10 min. Then, the mixture was centrifuged, and the supernatant was poured into a new tube.

A total of 400 µL of chloroform was added to the supernatant, and the tube was capped and vigorously shaken for 30 s. The next step involved centrifuging the mixture and transferring the supernatant to another new tube (about 500 µL). The same volume of isopropanol was added to the tube containing the supernatant, and it was mixed by turning it upside down and placed at room temperature for 30 min. Then, the mixture was centrifuged, and the supernatant was discarded. With the RNA at the bottom of the tube, 1 mL of 75% ethanol in DEPC was added to wash the RNA sediment. Next, the tube was centrifuged at 4 °C and 8000× *g* for 5 min, and the washing lipid was poured. A total of 10 µL of RNAse-free water was added to dissolve the cleaned RNA after it was washed twice. Then, 1 µL of RNA suspension was drafted on the nucleic acid quantifier to determine the concentration and purity of the extracted RNA. The 1% agarose gel electrophoresis technique was used to detect the integrity of total RNA extracted from breast milk. cDNA was prepared using a transcription kit (Vazyme, Nanjing, China) according to the manufacturer’s protocol.

### 2.5. Quantitative Real-Time PCR

The Fast Start Universal SYBR Green Master Mix (Roche) was used to carry out the quantitative real-time PCR experiment on the Agilent Stratagene MxPro system following the fast PCR protocol. The human glyceraldehyde 3-phosphate dehydrogenase (*GAPDH*) gene was selected as the internal reference for all analyses (No. B661104-0001, Sangon Biotech, Shanghai, China). Primers of *FADS1*, *FADS2* and *FADS3* genes were designed using NCBI Primer online, and the sequences are shown in Table 1.

### 2.6. Statistical Analysis

The basic information questionnaire database was established using Epidata 3.0 software. The mean ± standard deviation was used to express values of normally distributed data, the median (P25, P75) was used to express skewed data, and numbers (percentage) were used to express categorical data. SPSS Statistics software (version 24.0) and GraphPad Prism (version 7.0) were used to analyze the differences in PUFA levels in breast milk between different *FADS* gene transcription and translation groups. All statistical tests were two-tailed, and *p* < 0.05 was considered significant.

## 3. Results

### 3.1. Maternal Demographics of Participants

The maternal and infant demographics are shown in Table 2. The mean age of the 50 lactating women was 29.58 ± 2.42 years, and more than half of the participants were from middle-income households (52.00%). The mean preconception body mass index (BMI) of women was 20.68 ± 2.85, and they gained an average of 16.23 ± 5.53 kg of weight during pregnancy. More than half (52.00%) of the participants had a natural delivery. With respect to infants, their mean gestational age was 39.58 ± 0.93 weeks, and the mean birth weight was 3.45 ± 0.49 kg. A total of 72.00% of babies were exclusively breastfed, while the remaining received mixed feeding.

### 3.2. PUFA Profile of Breast Milk

The average levels of eight types of PUFA in breast milk detected in this study are listed in Table 3, including four types of n-6-series PUFA and four of n-3-series PUFA. Among the n-6-series PUFA, arachidonic acid (AA) accounted for 0.8616% of the total FAs. Among the n-3-series PUFA, the contents of EPA and DHA in breast milk were 0.1097% and 0.2952%, respectively.

### 3.3. Groups Divided by the mRNA Expression of FADS Gene

Groups were divided based on the median of mRNA relative expression levels among different isoforms of *FADS* genes, and depending on the expression level of each *FADS* gene, two more groups were created, as shown in Table 4. mRNA Q1 represents the low mRNA expression group, and mRNA Q2 represents the high mRNA expression group of each *FADS* gene. The expression of the three *FADS* genes was significantly different between groups (*p* < 0.001).

### 3.4. n-6 PUFA and the mRNA Expression of FADS Gene

**Figure 1:**
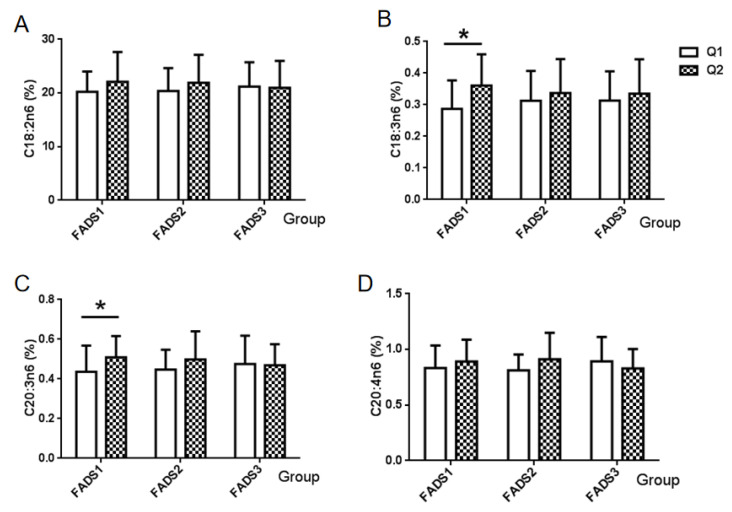
Association between the n-6 PUFA and mRNA expression groups of FADS genes. Values are the proportion of each n-6 PUFA of breast milk among FADS gene expression groups. (**A**) α-Linoleic acid (C18:2n6), (**B**) γ-linolenic acid (C18:3n6), (**C**) eicosatrienoic acid (C20:3n6), and (**D**) arachidonic acid AA (C20:4n6). * *p* < 0.05, Q1: Low mRNA expression group, Q2: High mRNA expression group.

The association between the mRNA expression of the *FADS* gene and four types of n-6 PUFA is shown in Figure 1. The amount of LA (C18:2n6) exhibited no differences among the mRNA expression groups of the *FADS1*, *FADS2*, and *FADS3* genes (Figure 1A). However, there were significant differences in γ-linolenic acid (C18:3n6) and eicosatrienoic acid (C20:3n6) among the *FADS1* gene mRNA groups (*p* = 0.009, *p* = 0.042, respectively). Furthermore, the level of γ-linolenic acid (C18:3n6) in *FADS1* mRNA group Q2 (0.3606%) was higher than that in *FADS1* mRNA group Q1 (0.2869%), which was also the case for eicosatrienoic acid (C20:3n6, Q1:0.5088% vs. Q2:0.4363%). No significant differences in these two types of n-6 PUFA were observed between the *FADS2* and *FAD3* groups (Figure 1B,C). Furthermore, no statistically significant differences were found in the proportion of AA (C20:4n6) in breast milk among *FADS* gene expression groups (Figure 1D).

### 3.5. n-3 PUFA and the mRNA Expression of FADS Gene

**Figure 2:**
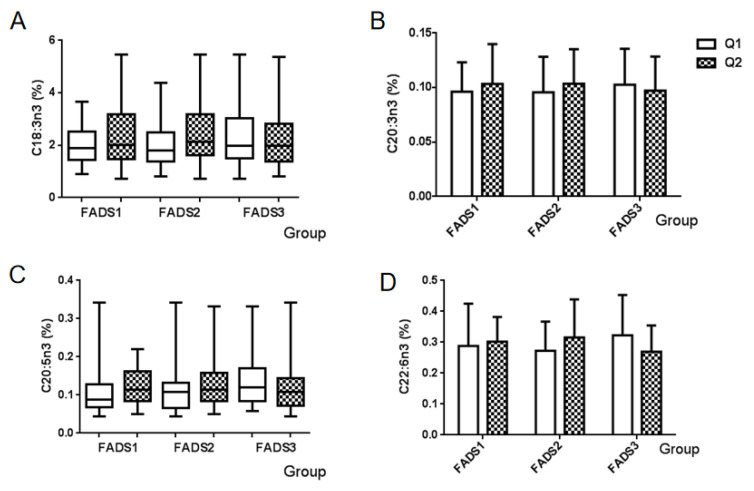
Association between the n-3 PUFA and mRNA expression groups of FADS genes. Values are the proportion of each n-3 PUFA of breast milk among FADS gene expression groups. (**A**) α-Linolenic acid (C18:3n3), (**B**) eicosatrienoic acid (C20:3n3), (**C**) EPA (C20:5n3), and (**D**) DHA (C22:6n3). Q1: Low mRNA expression group, Q2: High mRNA expression group.

The percentage of four types of n-3 PUFA in breast milk among the mRNA expression groups of the *FADS* gene is summarized in Figure 2. No significant differences were observed in all n-3 PUFA among *FADS* gene expression groups, including α-linolenic acid (C18:3n3), eicosatrienoic acid (C20:3n3), EPA (C20:5n3), and DHA (C22:6n3).

### 3.6. Enzyme Activity and PUFA Pathway Activity

**Figure 3:**
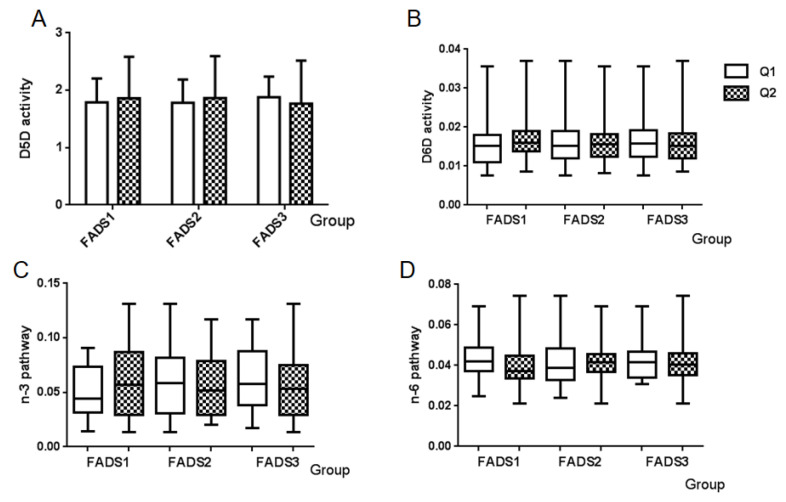
Distributions of enzyme activity and PUFA pathway activity among FADS gene expression groups. (**A**) D5D activity, (**B**) D6D activity, (**C**) n-3 PUFA pathway, and (**D**) n-6 PUFA pathway. Q1: Low mRNA expression group, Q2: High mRNA expression group.

Enzyme activity and PUFA pathway activity could be evaluated using the ratios between the product and precursor FA in breast milk. Enzyme activity included Δ-5 desaturase and Δ-6 desaturase activities, while PUFA pathway activity consisted of n-3 PUFA pathway and n-6 PUFA pathway activities. Calculation formulas [31] and proportions of these four indexes are summarized in Table 5. In this study, the proportion of Δ-5 desaturase activity was 1.8482, and that of Δ-6 desaturase activity was 0.0154; the n-3 PUFA pathway was 0.0554, and the n-6 PUFA pathway was 0.0418. Distributions of the four indexes among *FADS* gene expression groups are shown in Figure 3. No significant differences were observed in neither desaturase activity (Figure 3A,B) nor in the two PUFA pathways (Figure 3C,D).

## 4. Discussion

A total of 50 lactating women 42 to 45 days postpartum were enrolled in our study. Breast milk was collected to detect the FA profile and PUFA content. Moreover, the experimental protocol of RNA extraction from breast milk was established in this study. Furthermore, the mRNA expression levels in human milk among three *FADS* gene groups were detected by using the molecular biological technique. We conducted this study to explore whether the mRNA expression among *FADS* genes affects PUFA content of breast milk.

The average content of DHA in the breast milk of the participants included in this study was 0.2952%, which was lower than the corresponding global average content of 0.32% [32]. One of the main reasons for this observation is that Changchun City is located in the inlands of northeast China, and the intake of deep-water fish among lactating women is lower than that in the mothers from coastlands [21]. This led to the lower DHA content in breast milk in this study, while it was similar to that in 0.30% of the mothers from the North European country Latvia, where the consumption of fish among lactating women is low [33]. However, the average DHA content in breast milk in this research was lower than the average content of DHA in two populations of lactating women from China (0.36%) and Korea (0.67%) [21,34]. Furthermore, the average content of EPA in our study was 0.1097%, and it was also lower than that in Korean participants. The most probable reason for this result is that the breastfeeding women enrolled in this study were not taking PUFA supplements during lactation. Since PUFA supplements could affect the activity of key enzymes in PUFA metabolic pathways [35,36], participants who did not take PUFA supplements could rule out the effect of dietary supplements on the gene expression of key enzymes and provide better foundations for research on the relationship between breast milk PUFA and *FADS* gene expression. The average content of AA was the opposite to that of DHA and AA in breast milk in this study, and it was 0.8616% of the total FA and higher than that in Korean mothers (0.48%). It was also higher than the global average content (0.71%). One of the reasons might be that the main source of AA in the body is the conversion of its precursor substance LA, while the main source of LA in the lactating woman body is the plant-sourced oils, such as corn and peanut oils. The amount of plant-sourced oil intake among Chinese lactating women is high due to traditional cooking [37]. The large LA intake, together with n-6 PUFA derived from LA, could contribute to the high level of LA in the body, which competes with key enzymes of FA-synthesizing pathways; thus, inhibiting precursor substance of n-3 PUFA derivative for EPA and DHA [38]. Therefore, the high level of LA in the body among lactating women results in the high level of LA with n-6 PUFA derived from LA in their milk, which reduces the DHA concentration in breast milk. Therefore, the high percentage of AA in this study might be associated with the low EPA and DHA in breast milk [39].

Recent research has shown an association between *FADS* genes and FA desaturase activities [31]. We focused on four indexes to clarify the correlation between *FADS* gene expression and FA metabolism in breast milk. The four indexes of breast milk included Δ-5 FA desaturase and Δ-6 FA desaturase activities, as well as n-3 PUFA pathway and n-6 PUFA pathway activities. In our study, the level of Δ-5 FA desaturase activity was 1.8482, and that of Δ-6 FA desaturase activity was 0.0154. Few studies have focused on FA desaturase activity and PUFA pathway activity in breast milk. Research on FA desaturase activity in human plasma showed that the level of Δ-5 desaturase activity was 4.39, and that of Δ-6 desaturase activity was 0.0016 [40]. This indicates that the level of Δ-6 FA desaturase activity in breast milk in this study is similar to that of Δ-6 desaturase activity in plasma, while the level of Δ-5 FA desaturase activity in breast milk is lower than that in plasma. The average level of n-3 PUFA pathway activity was 0.0554, and that of n-6 PUFA pathway activity was 0.0418 in breast milk in our study. n-3 PUFA pathway activity is 0.842–1.001, and that of n-6 PUFA pathway activity is 0.233–0.389 in plasma. These results suggest the two PUFA pathway activities in human milk might be lower than those in plasma [31].

In the evaluation of the association between the PUFA of breast milk and the mRNA expression among *FADS* genes, we found that two types of n-6 PUFA were associated with the mRNA expression of the *FADS1* gene. GLA and eicosatrienoic acid (C20:3n6) in breast milk in the high mRNA *FADS1* gene expression group were higher than those in the other group. LA is the essential FA ingested from food, and it is the precursor substance for AA biosynthesis in our body. As a consequence, the LA proportion of breast milk is mainly derived from the food intake of lactating mothers. No significant differences in LA in breast milk among the mRNA expression groups of the three *FADS* genes demonstrated the consistent amount of LA sourced food intake among our participants. A recent study showed that LA content in the breast milk of Chinese mothers was positively correlated with the consumption of soybeans and soybean products [37]. According to a previous dietary survey by our research team [41], the amount of dietary intake LA was higher than its adequate intake [42]. Consequently, the amount of LA from dietary intake is sufficient to meet the level of LA in breast milk that satisfies the demands of infant growth and development.

The higher percentage of GLA in breast milk in the high *FADS1* gene *mRNA* expression group might be related to the higher level of transcription regulation of the *FADS1* gene among lactating women. Research demonstrated that the demand for PUFA was increased during pregnancy and lactation in women; thus, the dietary intake of FA by the mothers could activate the mechanism of gene transcription. Dietary PUFA and its metabolites act on the cellular nucleus level and regulate the mRNA expression of the *FADS* gene together with transcription factors [24]. An animal study indicated that suckling piglets that suckled milk from LA-supplemented sows showed increased mRNA expression levels of genes than those that suckled milk from palmitic acid-supplemented sows during gestation to weaning [43]. This study made it abundantly clear that dietary n-6-series PUFA intake was related to the mRNA level of gene expression.

Another study on rats showed significant overexpression of *FADS1* and *FADS2* mRNA during pregnancy and lactation in the breast, liver, and adipose tissues in low lipid diet fed rats compared with those fed an appropriate lipid diet [44]. The result of this research seems to contradict that of our study; however, it has been shown that lactating mothers have higher levels of LA intake based on previous studies in this article, which exceed the recommended intake level. Therefore, we conclude that when the level of the substrate LA in the body is higher, it could fully combine with key enzymes in pathways of PUFA metabolism and promote the downward conversion of the substrate LA to GLA, with the final product being AA. Gene expression plays a relatively small role when the dietary intake of lactating women is high. Consequently, there was no difference in the amount of AA in breast milk in this study among the mRNA expression groups of the three *FADS* genes. While the limited number of key enzymes was combined with enough substrate, the insufficient amounts of enzymes might promote the increase in the mRNA expression level of the *FADS* gene that synthesizes the key enzymes. Therefore, the higher percentage of GLA and eicosatrienoic acid (C20:3n6) in the high *FADS1* gene mRNA expression group might be affected by the interaction between maternal gene expression and dietary intake [45]. Once a certain level of the substrate AA is produced, a negative feedback regulation may occur. Δ-5 desaturase encoded by the *FADS1* gene is located closer to the substrate AA in the PUFA conversion pathway, while Δ-6 desaturase encoded by the *FADS2* gene is located farther away from the substrate AA [46]. The high level of substrate AA might inhibit the expression of *FADS1* genes that are closer to AA. Therefore, we only found the difference in the two types of n-6 PUFA intermediate products between the mRNA expression groups of the *FADS1* gene, while we did not find such a difference between the mRNA expression groups of the *FADS2* gene. We did not detect the association between any mRNA expression groups of *FADS* genes and n-3-series PUFA in this study. We infer that n-3 PUFA might be related to the protein expression level of the *FADS* gene cluster, and future studies need to verify our inference. There were no associations between *FADS3* gene expression and PUFA in breast milk in our study, and the function of the *FADS3* gene in relation to PUFA synthesis needs to be addressed in future studies [47,48].

We used human milk as the sample in this study to detect the association between mRNA expression of *FADS* genes and PUFA profiles in breast milk. The result of our study demonstrated that n-6 PUFA was associated with the mRNA expression levels of the FADS1 gene. To our knowledge, this is the first research in China to find the association between FADS gene expression and the PUFA in the breast milk of lactating mothers. While this study is innovative, it also has limitations. We did not collect the data on the dietary background of PUFA levels in the 50 lactating mothers. We only focused on the relationship between the level of PUFA and the *FADS* gene expression in breast milk, because it was impossible to evaluate the combined effect of maternal dietary intake and *FADS* gene expression on the PUFA profile in breast milk. According to the previous dietary survey by our research team on the maternal PUFA intake, we found that the intake of the essential FAs LA and ALA by lactating mothers far exceeded the reference intake, while that of EPA and DHA was far below the reference intake among our participants. However, the lack of differences in the content of these FAs among *FADS* gene mRNA expression groups indicated that the dietary intake in lactating women is relatively consistent. Therefore, the dietary PUFA in the included 50 lactating women might not have a significant impact on the results of this study. Furthermore, the milk samples analyzed in this study were collected from the 50 lactating women who were 42–45 days postpartum, which failed to reach the sample size for detecting genetic variants of some key single-nucleotide polymorphisms (SNPs) [22,28,49,50,51]. As a result, we could not combine *FADS* gene variants with their gene expression patterns and the PUFA profile in breast milk. A study has shown that *FADS1* gene polymorphism could regulate gene transcription in human liver tissue, thereby changing the composition of FA in the liver [52]. In our future studies, we shall continue to collect participants to reach the relevant number of samples necessary to detect key SNPs of *FADS* genes associated with PUFA profiles in breast milk to clarify the role of different SNP genotypes in *FADS* gene expression. Our study will provide a basis for guiding individual breastfeeding mothers with different genotypes to nurture infants scientifically and contribute to precision nutrition strategies.

## Figures and Tables

**Table 1:** Primer sequences of FADS genes used for quantitative RT-PCR amplification.

Gene	Primer	Sequence 5′→3′
*FADS1*	Forward	GTGGC TAGTG ATCGA CCGTA A
	Reverse	ATTCT TGGTG GGCTC AAAGC
*FADS2*	Forward	AACTG GTGGA ATCAT CGCCA
	Reverse	ATTCG CCCAG AACAA ACACG
*FADS3*	Forward	TCGTG ATGGG GCAGC TAAAG
	Reverse	CTTCT TGCCA TACTC GACGG A

**Table 2:** Maternal and infant demographics of the participants.

Characteristics	Mean ± SD/N (%)
Maternal age (year)	29.58 ± 2.42
Preconception BMI	20.68 ± 2.85
Gestational weight gain (kg)	16.23 ± 5.53
Gestational age (weeks)	39.58 ± 0.93
Birth weight (kg)	3.45 ± 0.49
Birth length (cm)	50.59 ± 1.26
Family income (yuan/month)	
<5000	9 (18.00)
5000–9999	26 (52.00)
≥10,000	15 (30.00)
Mode of delivery	
Vaginal	26 (52.00)
Cesarean	24 (48.00)
Feeding patterns	
Breastfeeding	36 (72.00)
Mixed feeding	14 (28.00)

**Table 3:** PUFA profile of breast milk in this study.

Series	PUFA		Percentage of Total FA (%) (Mean ± SD/Median (P25, P75))
n-6	Linoleic acid (LA)	C18:2n6	21.0886 ± 4.7437
	γ-Linolenic acid	C18:3n6	0.3245 ± 0.1008
	Eicosatrienoic acid	C20:3n6	0.4726 ± 0.1245
	Arachidonic acid (AA)	C20:4n6	0.8616 ± 0.1996
n-3	α-Linolenic acid (ALA)	C18:3n3	1.9504 (1.4444, 2.7370)
	Eicosatrienoic acid	C20:3n3	0.0999 ± 0.0320
	Eicosapentaenoic acid (EPA)	C20:5n3	0.1097 (0.0787, 0.1499)
	Docosahexaenoic acid (DHA)	C22:6n3	0.2952 ± 0.1114

**Table 4:** Relative levels of mRNA and groups divided by expression of *FADS* genes.

Gene	Relative Level of mRNA Median (P25, P75)	Group		*p*
		mRNA Q1	mRNA Q2	
FADS1	−0.1302 (−0.1319, −0.1255)	≤−0.1302	>−0.1302	<0.001
FADS2	−0.2619 (−0.3497, −0.1359)	≤−0.2619	>−0.2619	<0.001
FADS3	−0.5039 (−0.8220, −0.0132)	≤−0.5039	>−0.5039	<0.001

**Table 5:** Indexes of PUFA with breast milk in this study.

Index	Calculation Formula	Mean ± SD/Median (P25, P75)
D5D *	C20:4n6/C20:3n6	1.8482 ± 0.5725
D6D *	C18:3n6/C18:2n6	0.0154 (0.0127, 0.0190)
n-3 pathway	C20:5n3/C18:3n3	0.0554 (0.0303, 0.0798)
n-6 pathway	C20:4n6/C18:2n6	0.0418 (0.0354, 0.0481)

* D5D: Δ-5 desaturase activity; D6D: Δ-6 desaturase activity.

## Data Availability

All the data of our research are reported in this manuscript, and they are available from the corresponding author.
